# Delusions and prediction error: clarifying the roles of behavioural and brain responses

**DOI:** 10.1080/13546805.2014.990625

**Published:** 2015-01-03

**Authors:** Philip Robert Corlett, Paul Charles Fletcher

**Affiliations:** ^a^ Department of Psychiatry, Ribicoff Research Facility, Yale University, 34 Park Street, New Haven, CT, USA; ^b^ Department of Psychiatry, School of Clinical Medicine, University of Cambridge, Addenbrooke's Hospital, Hills Road, CambridgeCB2 0SP, UK; ^c^ Department of Psychiatry, Cambridgeshire and Peterborough Mental Health Partnership NHS Trust, CambridgeCB1 5EE, UK

**Keywords:** delusions, prediction error, functional neuroimaging, cognitive neuroscience

## Abstract

Griffiths and colleagues provided a clear and thoughtful review of the prediction error model of delusion formation [Cognitive Neuropsychiatry, 2014 April 4 (Epub ahead of print)]. As well as reviewing the central ideas and concluding that the existing evidence base is broadly supportive of the model, they provide a detailed critique of some of the experiments that we have performed to study it. Though they conclude that the shortcomings that they identify in these experiments do not fundamentally challenge the prediction error model, we nevertheless respond to these criticisms. We begin by providing a more detailed outline of the model itself as there are certain important aspects of it that were not covered in their review. We then respond to their specific criticisms of the empirical evidence. We defend the neuroimaging contrasts that we used to explore this model of psychosis arguing that, while any single contrast entails some ambiguity, our assumptions have been justified by our extensive background work before and since.

We were pleased to see Griffiths, Langdon, Le Pelley, and Coltheart (2014) review the evidence that abnormal prediction error may be important in delusion formation. We agree with their overall conclusion that there is a growing evidence base supporting the idea and we are pleased that they share our enthusiasm for applying an associative learning framework to understanding psychosis. Such a framework offers a set of testable hypotheses about how disturbances in basic learning processes may lead ultimately to altered models of the world and, hence to profound shifts in the ways in which sensory evidence is processed, attended to and interpreted (Corlett, Honey, & Fletcher, 2007). Furthermore, concepts derived from associative learning offer a level of analysis and description that will prove very useful in the quest to link disturbed brain processes to alterations in cognition and experience (Corlett, Frith, & Fletcher, 2009; Corlett, Honey, Krystal, & Fletcher, 2010). Moreover, key concepts that describe associative learning fit well with computational perspectives on perception, inference and decision-making (Friston, 2005; Friston & Stephan, 2007). We believe that such computational psychiatry approaches (Corlett & Fletcher, 2014; Friston, Stephan, Montague, & Dolan, 2014; Montague, Dolan, Friston, & Dayan, 2012; Stephan & Mathys, 2014) offer the opportunity to move beyond the useful but necessarily limited and often metaphorical accounts that shape many cognitive accounts of delusions.

We feel that it is useful to add some comments to the paper of Griffiths et al.: first, we would like to offer some refinements to the prediction error account of delusions and to embed it more thoroughly in hierarchical models of perception and inference (Friston, 2005; Friston & Stephan, 2007). We believe that this is important in demonstrating its potential value in linking what we know about brain function with higher-level descriptions that shape our understanding of psychopathology (Corlett et al., 2009, 2010). Second, we underline what we consider to be an important characteristic of this model – that characterisation of disruption in prediction error signal, as well as providing a parsimonious and comprehensive account of how delusions emerge, may help explain their resistance to contradictory evidence, their elasticity and the characteristic co-occurrence of perceptual anomalies (Corlett et al., 2013; Corlett, Krystal, Taylor, & Fletcher, 2009). Third, we wish to respond to the critique of our work (Corlett et al., 2004, 2006, 2007) that Griffiths et al. presented in the second part of their paper.

## A fuller perspective on the prediction error model

### Challenging the perception-belief dichotomy

Models of delusional belief have tended to be expressed in ways that implicitly or explicitly treat perception and inference as fundamentally separable phenomena, a dichotomy that has led to some contention over whether delusions emerge from normal inferences acting on abnormal perceptual experiences (Kapur, 2003; Maher, 1974, 1988) or abnormal inferences acting on normal experiences (Campbell, 2001; Currie, 2000). It has been argued that neither deficit alone could account for a delusional belief and that two factors – both abnormal perception and abnormal inference (in this case the ability to evaluate beliefs, which is of course itself an inference) – must be disturbed (Coltheart, 2010; Coltheart & Davies, 2000). While the latter is a cogent argument, we believe that it is only necessary in so far as there is a clear distinction between perception and inference: a distinction which is not actually compatible with what is known about how the brain deals with the world (Barlow, 1990; Helmholtz, 1878/1971). Increasingly, influential views consider the brain as a predictive device that makes inferences about the world (Friston, 2005; Friston & Stephan, 2007). Specifically, it must estimate the likely cause of an input, a process that may be known as abductive inference (Peirce, 1931–1958) and, notably, one that Coltheart has pointed out is characteristic of delusional beliefs (Coltheart, Menzies, & Sutton, 2010). This is actually an insoluble problem of inference and the best that the brain – isolated as it is from the reality of the world – can hope for is an informed guess. The abductive guess is informed by prior experience, which is of course the essence of Bayesian processing.

So the simple idea is that a perception even at the lowest levels is actually shaped by what is already known – it is an inference about the cause of a sensation and cannot be readily separated from belief (Friston, 2005; Friston & Stephan, 2007). Experience enables predictions that shape inference (Friston, 2005; Friston & Stephan, 2007). In a system that is arranged hierarchically, we may perhaps choose to refer to the inferences at the lower levels as perceptions and the inferences at the higher levels, being more abstract and immutable, as beliefs, but we suggest that it is important to consider that similar processing pertains at all levels of the hierarchy – upcoming signal is compared with current predictions (Friston, 2005; Friston & Stephan, 2007). The ensuing experience is a consequence of the brain striving to find the prediction that best fits the signal (Friston, 2005; Friston & Stephan, 2007). A prediction that fails to account for current input leads to a prediction error signal which, depending on its nature, may either be suppressed or may percolate to higher levels in the hierarchy where it may ultimately (though not necessarily) be the drive towards new predictions – that is, new beliefs (Friston, 2005; Friston & Stephan, 2007). Note too that this model incorporates inferences about inferences: an inference at one level that violates the expectations embodied at a higher level will generate a prediction error which again may be suppressed or may lead to an alteration in those expectations. This could be equated to belief evaluation.

From this perspective, a dispute about whether delusions arise from abnormal experiences, abnormal inferences or both becomes unhelpful and potentially meaningless. Indeed, it might be argued that the treatment of delusions and hallucinations as distinct entities is also fundamentally challenged by this insight. In essence, we are suggesting that, although it is possible – and sensible – at one level of analysis to distinguish beliefs from perceptions (and delusions from hallucinations) at another level of analysis – the one that we think is more useful – no distinction is called for (Friston, 2005; Friston & Stephan, 2007). We believe that this is an important point to make. While Griffiths et al. are very clear that the prediction error model is a single deficit model of psychosis, we wish to add that the employment of a hierarchical predictive coding model points to the important principle that perceptions and beliefs (and, by implication, hallucinations and delusion) should not, indeed cannot at certain key levels of description, be separated. That is, we are not talking simply about a single deficit that affects two qualitatively distinct sets of mental processes but rather invoking a model that points to deep similarities between perception and belief.

### More than just the emergence of a new belief

A further point that we think should be emphasised and, one that has been developed more recently, is that, although altered prediction error may most directly and clearly account for the emergence of delusions, insights from associative learning studies actually show how the argument may be extended to account for the fact that delusions become strongly fixed and, at the same time, sufficiently elastic to incorporate new evidence – even evidence that seems directly contradictory to the core of the belief.

We suggest that the process through which beliefs are relinquished and replaced with alternative beliefs could be likened to extinction learning (Corlett et al., 2009). Extinction learning is invoked when a previously reinforced association is reinforced no longer (Bouton, 2000). New, context-dependent, learning ensues, learning not to expect reinforcement (Bouton, 2000). The interplay between new and old learned expectations is delicate and it may be modulated by prediction error (Eisenhardt & Menzel, 2007). In a study of fear memories in crabs, definitively confounding the crab's learned expectation engendered extinction learning. However, reactivation of the learned expectation without disconfirming it (reminding crab of the reinforced situation) actually strengthened the memory (Pedreira, Perez-Cuesta, & Maldonado, 2004). That is, a surprising reminder of a reinforced situation strengthens the memory for that situation, even when the reinforcement does not occur (Eisenhardt & Menzel, 2007; Pedreira et al., 2004). This effect has been reported in rodents (Lee, 2008). The importance of prediction error in human memory reconsolidation has also been confirmed (Sevenster, Beckers, & Kindt, 2012, 2013). We have argued that aberrant prediction errors drive delusion formation (Corlett, Taylor, Wang, Fletcher, & Krystal, 2010). If prediction errors also drive memory strengthening, then aberrant prediction errors ought to entail aberrant memory strengthening (Corlett, Taylor, Wang, Fletcher, Krystal, 2010).

We can express this in terms of the predictive coding account too. If delusions form as new “priors” as a consequence of altered prediction error signal, then it is the nature of the hierarchical predictive system that they are deployed to predict and explain future experiences. Critically, the delusion has formed as the best way to account for a noisy and uncertain prediction error [and one that perhaps has an unjustly elevated level of precision (Adams, Stephan, Brown, Frith, & Friston, 2013; Corlett et al., 2010; Fletcher & Frith, 2008)]. But it is unlikely to be very successful in accommodating this error signal. Over time, a haphazard error signal – even a strong one – will become discounted – it will not contribute to updating (Preuschoff & Bossaerts, 2007). Thus, the reactivation of the prior, even though it does not accurately predict the ensuing input, will have a reinforcing effect on that belief. Similar, so-called *backfire,* effects are observed in politics (Bullock, 2009) and science (McRaney, 2013). Indeed, ketamine – an experimental model of psychosis – may produce a comparable effect (Corlett et al., 2013).

## Clarifying the role of prediction error in psychosis

We feel that the neural responses in our studies are best described as aberrant prediction errors, errors in response to events that really ought not to be surprising. This pattern of excessive responses to unsurprising events is present in all of our studies [and in other studies that do not explicitly manipulate prediction error (see Anticevic & Corlett, 2012 for a review)]. It is probably most unambiguously demonstrated in our study of forward blocking in healthy individuals with schizotypal beliefs (e.g. beliefs in telekinesis or alien abduction). Here, subjects learned that one cue (e.g. apples) predicted the allergy (A+). In a subsequent phase of training, they learned that apples and bananas predicted the same allergy (AB+). The outcome was already fully predicted by the apple (A), and there should have been no prediction error on AB+ trials and hence nothing should have been learned about B.

**Figure 1.**
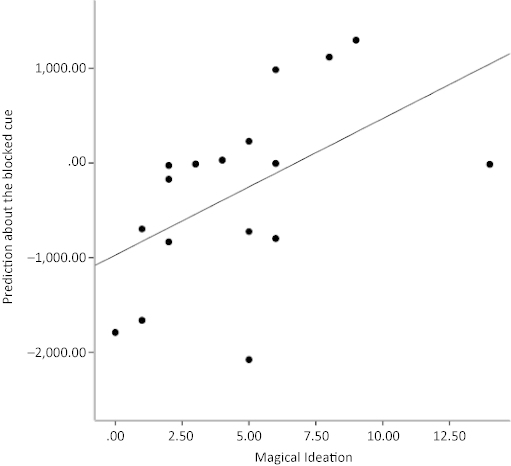
Relating behavioural predictions to delusion-like ideas. Note: Scatterplot depicting the relationship between subjects' behavioural predictions about the blocked cue and their self-reported magical ideation measured with the Chapman scale (Eckblad & Chapman, 1983).

When we examined brain responses to AB trials, we found that on average, dorsolateral prefrontal cortex (DLPFC) was less active than on control trials (Stage 1, C–, Stage 2, CD+). However, some subjects engaged DLPFC (and hence prediction error signalling) more than others in response to AB+ trials. This was manifest as behavioural learning about B, subjects with inappropriate prediction error responses at Stage 2, learned that B caused the allergy (Corlett & Fletcher, 2012). In response to Griffiths et al.'s concerns that we do not discuss behavioural data sufficiently, we returned to this data-set, examining the relationship between this inappropriate learning about redundant stimuli and the extent of subjects' odd beliefs (as measured by the magical ideation subscale scores from the Chapman Schizotypy scale). There was a significant correlation between subject's learning about the redundant stimuli (B?) and their odd-beliefs (*n* = 17, *r* = 0.5, *p* = 0.03, see Figure 1). We take Griffiths et al.'s point here, clearly simpler designs are easier to communicate and it is easier to use them to link inappropriate prediction error brain signal to delusion-like ideation unambiguously.

## Considering Griffiths et al.'s critique of retrospective revaluation studies

Now, we turn to the more specific critique of our work and the evidence that it provides for the prediction error model of delusions. First, we are grateful to Griffiths and colleagues for their careful consideration of the experiments and for setting out the key components so clearly. We should acknowledge that we do not hold a monopoly on providing the relevant experimental evidence and there are other examples of cortical (Schlagenhauf et al., 2009; Schmack et al., 2013) and subcortical prediction error signals (Romaniuk et al., 2010) that are inappropriately engaged in people with delusions. We are glad too that their central conclusion is that aberrant prediction error has promise as an explanatory mechanism for delusion formation. But we are naturally keen to defend the work against some of the criticisms levelled at it.

Griffiths et al. have three main criticisms of our work:

1. We do not show a retrospective revaluation effect nor is behaviour on the task disrupted by psychosis.
2. We use reverse inference to make the case that what we are observing in DLPFC is a prediction error signal.
3. We did not choose the best trial types from our design to identify prediction error signals.

These three concerns really boil down to one important question in the context: Were we able to identify neural responses specific to expectancy violation (i.e. prediction error) and to relate variations in these responses to psychosis. This is less relevant to one of the paper that is discussed (Corlett et al., 2004) which was actually using a brain response as the basis for interpreting the impact of the associative learning manipulation. In this study, we made certain key observations that formed the basis for interpreting the findings of aberrant right prefrontal cortex (rPFC) activity in psychosis and ketamine. First, we showed that a specific focus of rPFC activation – one that we had shown in prior causal associative learning studies to code prediction error – accompanied the retrospective revaluation manipulation lending support to the modified associative account of revaluation. The fact that there was a strong, though not significant, trend towards behavioural report of altered predictive strength at a later stage we took to be evidence that such revaluation was occurring. Griffiths et al. argue that we were incorrect to do this. We respond that such behavioural measures may not have been sufficiently sensitive to reflect a genuine revaluation effect and point to another finding in this paper: That the degree of subject-specific rPFC activation during expectancy violation in the final stage was predicted by the degree of activation occurring at the prior revaluation stage (Stage 2). This strongly suggested to us that Stage 2 involved processes that related directly to updating expectancies. We argue that, for such an experiment, behavioural data are noisier and less sensitive than brain imaging data. We recognise that this is a speculation and acknowledge that Griffiths et al. do not believe it. The question of whether neuroimaging findings can be interpreted or believed in the absence of behavioural changes is reviewed carefully elsewhere (Wilkinson & Halligan, 2004).

Of course the above argument – and indeed the experimental design as a whole – did indeed involve so-called reverse inference. We defended this approach in the original paper and we continue to do so. There are of course problems with reverse inference. Merely observing a brain response in a particular region in a novel task (in say the striatum) does not entail a specific psychological process (e.g. reward) is occurring in that task. However, reverse inference is not always inherently flawed (Hutzler, 2014). If we take a Bayesian approach, given that subjects are doing the sort of causal learning task in which we have previously observed DLPFC responses that are co-incident with prediction error (e.g. superlearning, preventative learning and simple associative learning) and we now observe identical activation in a similar causal learning setting (retrospective revaluation), it is reasonable to infer that we are observing prediction error. Of course, we do not believe that all DLPFC BOLD responses are prediction errors; we never claimed that they were. What we have been striving for is a brain marker for the occurrence of prediction error signal. With such a marker we are in a position to test models of disrupted processing.

Most importantly, we turn to the comment in Griffiths et al.'s paper that “A serious shortcoming of the studies reported by Corlett et al. (2004, 2006, 2007) is that the control cues in the fMRI contrasts were not appropriate”. They point out, for example, that the trials of interest entail not merely prediction error but also incidental recall of cues with which the critical cues had previously been paired. We draw their attention to the methods section (Corlett et al., 2004) in which we describe how we subtracted out the effects of within-compound associations at both Stages 2 and 3 precisely as they suggest. Indeed, it was because the retrospective revaluation manipulation offered an elegant way of manipulating stimulus-related expectancy while controlling for associative history and structure that we first became interested in it. While we would argue that contrast used to identify prediction error-dependent updating in this experiment is the most tightly controlled that we have seen, the subtlety of the effect and the unreliability of the backward blocking effect meant that we used, when we carried out analyses of prediction error responses in patients and volunteers receiving ketamine, we used a lower level baseline. As Griffiths et al. point out the danger of a low-level baseline condition is that it may differ from the experimental condition in more just the process of interest. We argue, however, that the body of work leading up to these more clinical studies engenders sufficient confidence that the precise area of rPFC in the context of casual associative learning tasks correlates with prediction error-dependent updating and the fact that it showed clear group differences (including abnormally high levels of response to the low level baseline trials in patients and volunteers) under ketamine and that the magnitude of this disruption related to delusion-thinking. As an internal check (Corlett et al., 2007), we used a supplemental analysis in which we specifically explored, on a trial by trial basis, how the magnitude of violation related to rPFC activity using the actual predictions (modulated by confidence) that each participant had made for each trial. Again we found a group difference and found that this related to delusional thinking in patients.

To be clear, we cannot argue that, for any given comparison or study, we unequivocally demonstrate that group differences are characterised by prediction error abnormalities and nothing else. Indeed we have been working since on finding new ways of addressing this question. Some of the ways, including the use of forward blocking (Corlett & Fletcher, 2012, 2013), coincide with suggestions made by Griffiths et al. Moreover, while we do argue that these three studies strongly support a prediction error model of psychosis, we would present them not as a fait accompli but as an emerging narrative that has been sufficiently compelling for us to continue to pursue it.

Griffiths et al. suggest that the controls we chose and the regions we identified suggest that our findings may be interpreted in terms of perturbed working memory. We disagree, having shown that in the same participants (Corlett et al., 2006) tasks engaging working memory and attention associated with negative symptoms (Honey et al., 2008). Conversely, in the patient group, a separate reward learning task (Murray et al., 2007) was associated with altered prediction error responses.

However, Griffiths et al. do highlight an important inadequacy our choice of controls. The J cue ought to be less associable. That is, J should garner less attention and enter into fewer associative relationships. In prior work, Le Pelley, Griffiths and their colleagues have demonstrated that patients with schizophrenia (Morris, Griffiths, Le Pelley, & Weickert, 2013) and individuals with schizotypal personality traits (Le Pelley, Schmidt-Hansen, Harris, Lunter, & Morris, 2010) find irrelevant stimuli more associable during learning tasks. Prediction error is one mechanism through which cues garner subsequent associability (Courville, Daw, & Touretzky, 2006; Pearce & Hall, 1980). However, it is not only candidate process but individuals can also find highly predictive stimuli more associable (Mackintosh, 1975). Hybrid models that combine prediction error and associability have been proposed (Le Pelley, 2004).

Associability is difficult to examine in a neuroimaging setting using subtractive analyses. One study used computational modelling to generate trial-by-trial regressors for associability and prediction error (Li, Schiller, Schoenbaum, Phelps, & Daw, 2011; Roesch, Esber, Li, Daw, & Schoenbaum, 2012). Associability correlated with activity in the amygdala and anterior cingulate cortex [regions that also signal prediction errors (Chumbley et al., 2014; Holroyd & Coles, 2002; McHugh et al., 2014)]. Furthermore, Li et al. also observed an associability signal in DLPFC, head of caudate and midbrain – in regions that signal prediction error in our analyses (see Li et al., supplementary materials). Future work should try to dissociate associability from prediction error (which will be a challenge) and then explore the contribution of each to the genesis of delusions.

More broadly, this exchange points to a tension in cognitive neuroscience. It is not clear exactly how we can make bridges between cognitive and neural science. One approach, predicated on neuropsychology, is to assume that one region performs one function (hence, when that region is damaged, the process is lost – this is basis for neuropsychology using cognitive tasks to infer the location of brain damage). This is a sort of Swiss Army Knife approach to neuro-cognition – it is modular (specific tools solve particular problems) and limited (there are a finite number of tools). There are lesion patients whose cognitive dysfunctions support such mappings from specific functions to particular regions [although post-mortem data can sometimes call that specificity into question (Annese et al., 2014)]. It would seem that this mapping approach would support our assertion that DLPFC and prediction error are associated. It would also support the lesion-based account for delusions proposed by Coltheart and colleagues.

However, it does not support the observation of activations in similar regions when different processes are engaged (e.g. DLPFC is often engaged during working memory). Nor does it allow for the redundancy and capacity for recovery in the human brain. We suggest instead that cognition might be more meaningfully mapped to brain function in terms of distributed circuits; networks of interacting brain regions that dynamically reconfigure in response to different tasks (Cole et al., 2013). In our causal learning task, when prediction errors occur, the DLPFC is co-active with the head of the caudate and midbrain. In working-memory tasks, thalamus, cerebellum and parietal cortex are often co-activated with DLPFC. This is by no means exhaustive and task engendered circuits are not necessarily exclusive, however, in this way, we can explain how we can observe activations in similar brain regions across a range of apparently unrelated cognitive tasks. This dynamic reconfiguration in response to the task at hand is more like the T1000 robot in the movie Terminator 2. The machine is made from a liquid mimetic poly-alloy and can take on a range of different forms in order to solve new problems. Meta-analyses of task-based fMRI data will help us identify these circuits and compare them across tasks.

This discussion leads to a final point that we would like to make in response to Griffiths et al. Given this more dynamic relationship between brain activity and cognition, it does not seem appropriate to attribute regional responses to processes such as belief and perception nor to separate them on neuroanatomical grounds. Instead, the dynamic model allows for an influence of belief on perception and, vice versa, influences for which behavioural evidence abounds. We believe prediction error is reflected by regional activations and regional interactions. Delusions arise as a result of aberrant prediction errors at both of these levels of analysis. We agree with Griffiths et al. that the work on prediction error thus far is supportive of the model. And we are happy too with their proposal for robust experiments that may further test and refine the model. We look forward to following the outcomes of such experiments.
